# Feasibility analysis of total quality management in medical quality management and its impact on work efficiency

**DOI:** 10.3389/fmed.2025.1701801

**Published:** 2025-12-10

**Authors:** Peiyao Shi

**Affiliations:** Hospital Quality Management Office, Shenzhen Yantian District People’s Hospital, Shenzhen, Guangdong, China

**Keywords:** total quality management, medical quality management, work efficiency, medical service quality, safety quality, medical quality assessment

## Abstract

**Objective:**

This study aimed to analyze the feasibility of total quality management (TQM) in medical quality management (focusing on three dimensions: management work efficiency, medical service quality, and safety quality) and to evaluate its impact on the work efficiency of medical staff.

**Methods:**

TQM was introduced in January 2023, serving as the dividing point between the pre-management period (January-December 2022) and the post-management period (January-December 2023). Fifty medical staff members from the same cohort were chosen as study subjects. Comparisons were made between the pre- and post-management periods in terms of work efficiency (hospital culture development, reward and punishment mechanisms, communication and coordination mechanisms, environmental hygiene management mechanisms, and adverse event reporting mechanisms), medical service quality, staff satisfaction, safety quality (incidence of adverse events, unplanned returns to the operating room, qualification rate of medical device sterilization, incidence of infection events, and medical complaints), and overall medical quality (medical safety management, medical training management, and medical hygiene supervision). Data were analyzed using SPSS 29.0. Paired-sample *t*-tests were employed for continuous variables (scale scores), while χ^2^ tests were used to categorical variables (satisfaction, safety quality).

**Results:**

After TQM implementation, the work efficiency of medical staff significantly increased (*P* < 0.001). Compared to the pre-management period, the post-management period exhibited higher medical service quality scores, greater overall staff satisfaction, lower incidences of adverse events, unplanned returns to the operating room, infection events, and medical complaints, a higher sterilization qualification rate for medical device, and enhanced medical safety, training, and hygiene supervision management (all *P* < 0.05).

**Conclusion:**

TQM is feasible and effective in medical quality management. Its implementation can enhance work efficiency and medical service quality among medical staff, improve satisfaction, strengthen safety quality, and comprehensively elevate overall medical quality.

## Introduction

With the continuous advancement of medical technology and the ever-growing health demands of the population, the importance of medical quality management has become increasingly prominent. Medical institutions are under continuous pressure to maintain high-quality nursing services, control disease transmission, and achieve sustainable management (1). Ensuring patient safety, enhancing staff competence, and improving the quality of medical services are fundamental elements in building an efficient healthcare system, directly influencing the overall wellbeing and living standards of society (2). Therefore, exploring systematic and evidence-based approaches to quality management has become an urgent research focus in the field of hospital management.

In this context, total quality management (TQM), as an advanced managerial concept, has gradually gained attention along healthcare administrators (3). Since the 1990s, TQM has been widely applied across multiple industries in developed countries, emerging as a key trend in modern management practices (3). With continuous quality improvement as its core principle, the TQM framework emphasizes achieving quality enhancement, cost optimization, and service excellence through organization-wide participation and full-process management, thereby driving sustainable organizational development (2, 3). In sectors such as pharmaceuticals and food production, TQM has proven effective in embedding sustainable practices, minimizing resource wastage, and enhancing operational transparency—measures that strengthen customer trust and foster long-term partnerships (4). Although originally developed for the industrial field, the application of TQM has progressively expanded to public service domains such as healthcare and education (3). For instance, the introduction of TQM in educational institutions has substantially elevated management policy standards and achieved positive outcomes in areas including evaluation feedback, customer orientation, and leadership commitment (5).

Within healthcare systems, TQM functions as a structured, data-driven management model that aligns closely with the strategic objectives of medical institutions. It enhances process effectiveness, efficiency, and adaptability through a combination of qualitative and quantitative tools, maintaining a patient-centered focus and promoting a culture of continuous quality improvement (3, 6, 7). Evidence shows that applying TQM principles can improve patient care, reduce medical errors, and increase employee engagement and organizational commitment (3). Moreover, TQM has demonstrated significant clinical value in reducing needlestick injuries, as systematic process improvements effectively enhance nursing quality and safety (8). Furthermore, Majdi et al. further confirmed that TQM interventions can facilitate ongoing quality enhancement and contribute to the delivery of cost-effective, high-quality care (2).

Nevertheless, existing research on TQM in healthcare primarily concentrates on individual case summaries, leaving its applicability across diverse medical institutions and its mechanisms for influencing organizational operational efficiency insufficiently clarified. Against this background, the present study, grounded in the practical context of our hospital, aims to examine the impact of TQM on medical management efficiency, medical service quality, safety quality, and the work efficiency of healthcare personnel. Through an in-depth empirical analysis, this research seeks to provide evidence-based insights and practical guidance for advancing quality improvement strategies within specific medical settings.

## Materials and methods

### Ethical statement

This study was reviewed and approved by the Medical Ethics Committee of Shenzhen Yantian District People’s Hospital. Prior to study initiation, designated ethics liaisons from the research team provided detailed, one-on-one explanations to all potential participants regarding the study’s purpose, duration, procedures, and participant rights protection measures. These included assurances that research data would be used solely for academic analysis, anonymized during processing, and that all personal information would remain strictly confidential. Participants were informed that they could voluntarily withdraw from the study at any time without any impact on their normal work or associated benefits. After being fully informed, all participants voluntarily signed the Informed Consent Form and confirmed their willingness to participate in the study.

### Study subjects

The sample size for this study was calculated using G*Power version 3.1.9.2. With parameters set as Power = 0.85, α = 0.05, Effect size = 0.5, and Number of groups = 1, the minimum required sample size was determined to be 38 participants. Considering a potential 15% attrition rate, a total of 50 participants were ultimately included.

TQM implemention began in January 2023. Accordingly, the study period was divided into two phases: the pre-management period (January to December 2022) and the post-management period (January to December 2023). During these two periods, 50 medical staff members from the same cohort were recruited as study participants. Among them, there were 28 females and 22 males, aged 25–45 years, with a mean age of (35.76 ± 7.06) years. The working experience ranged from 7 to 15 years, with a mean of (10.38 ± 2.63) years.

Inclusion criteria: ① medical staff with at least 7 years of clinical experience; ② individuals with good compliance and the ability to cooperate with training and assessment. Exclusion criteria: ① those on standby or personal leave for more than 1 month; ② individuals who have resigned or were transferred to other positions; ③ pregnant or lactating women.

### Study tools

(1) Medical staff management work efficiency before and after management: A self-developed *Medical Staff Management Work Efficiency Assessment Scale* was used for evaluation, revised with reference to the *Key Points of Core Medical Quality and Safety Systems*. The scale comprised five core dimensions: hospital culture development (4 items), reward and punishment mechanisms (4 items), communication and coordination mechanisms (4 items), environmental hygiene management mechanisms (4 items), and adverse event reporting mechanisms (4 items), totaling 20 items. Each item was rated on a 5-point Likert scale (1 = completely inconsistent, 5 = completely consistent). Scores for each dimension were converted to a 100-point scale using the formula: (actual dimension score/maximum dimension score) × 100. Higher scores indicated better management quality in the corresponding dimension for medical staff. The Cronbach’s α coefficient for the scale was 0.87, indicating good internal consistency.

(2) Medical service quality: A self-developed *Medical Service Quality Assessment Scale* was employed for quantitative evaluation, revised based on the core requirements of clinical medical services with reference to the *Medical Quality Management Measures*. The scale included five key dimensions: clinical nursing and medical capabilities (5 items), interpersonal relationships (5 items), ethics and law (5 items), professional development (5 items), and critical thinking (5 items), totaling 25 items. Each item was rated on a 5-point Likert scale (1 = very poor, 2 = poor, 3 = average, 4 = good, 5 = excellent). Scores for each dimension were converted to a 0–20 point scale using the formula: (actual dimension score/maximum dimension score) × 20. Higher scores reflected better medical service quality in the corresponding dimension. The Cronbach’s α coefficient was 0.85, confirming acceptable reliability.

(3) Satisfaction: A self-developed *Medical Staff Job Satisfaction Assessment Scale* was used for evaluation, revised from the *Minnesota Satisfaction Questionnaire (MSQ) Short Form*. The scale contained four core dimensions with a total of 10 items: work environment and resource support (3 items), work process and efficiency (2 items), team collaboration and management support (2 items), and career development and incentive mechanisms (3 items). Each item was rated on a 10-point scale (1 = completely dissatisfied, 10 = completely satisfied). The total score represented the cumulative sum of the 10 items (maximum score = 100). Grading criteria: 80–100 points indicated very satisfied, 60–79 points indicated satisfied, and < 60 points indicated dissatisfied. Total satisfaction (%) = (number of very satisfied + satisfied cases)/total number of cases × 100%. The reliability and validity tests showed a Cronbach’s α coefficient of 0.82.

(4) Safety quality before and after management: Safety quality indicators included the incidence rate of adverse events, rate of unplanned return to the operating room, sterilization qualification rate of medical devices, incidence of infection events, and rate of medical complaints.

(5) Medical quality levels before and after management: Evaluation focused on three key dimensions: medical safety management (e.g., system implementation, risk prevention and control), medical training management (e.g., training coverage, pass rate of assessments), and medical health supervision management (e.g., compliant diagnosis and treatment, process standardization). A standardized hospital self-assessment procedure was adopted. The Quality Control Department led the formation of an assessment team comprising three clinical directors and two quality control specialists to implement uniform scoring standards. Each dimension had a maximum score of 20 points, with higher scores representing better quality. The inter-rater reliability intraclass correlation coefficient (ICC) was 0.86 (95% CI: 0.78–0.92). Data sources included self-assessment records and quality control ledgers to ensure the reliability of evaluations.

### Study process

(1) Pre-management. From January to December 2022, the hospital adopted a conventional quality management model. Internal quality management standards were developed, quality management guidelines were refined, hospital safety management was strengthened, and the emphasis on medical quality was reinforced. Relevant quality management documents and guidelines were issued and communicated within the hospital to ensure that all personnel were informed of the latest standards. All medical staff were required to study and understand these updated guidelines to maintain consistent implementation across departments.

(2) Post-management. Beginning in January 2023, the hospital implemented TQM. ① Establishment of a quality management team: The team leader was appointed by the hospital dean, with the director of the Quality Management Office serving as the executive deputy leader, and the directors of the Medical Affairs Department, Nursing Department, and Infection Control Department serving as deputy leaders. The team consisted of two outstanding representatives from each of the four departments mentioned above. The entire team systematically studied the hospital’s TQM standards to ensure comprehensive understanding and consistent execution. ② Improvement of regulatory systems: Based on the actual occurrence of adverse events, medical quality incidents, and patient admission situations, existing quality management measures were reviewed and refined. Medical quality and safety management objectives were formulated according to TQM principles, and relevant processes were established and improved. Through multiple channels and methods, all medical staff received training in TQM-related knowledge. Key personnel were organized to analyze problems identified during quality management implementation, propose corresponding solutions, and integrate them into practical management processes, thereby enhancing the overall level of medical quality and safety management in the hospital. ③ Enhancement of basic training: The hospital established a standardized educational system and collaborated with institutions that had successfully implemented TQM to formulate training requirements. Training content was organized, standardized, and submitted to the Medical Education Department for record-keeping. The training primarily focused on quality management concepts and professional expertise. Furthermore, vocational skills training for new employees and managerial personnel was strengthened. Regular educational programs were conducted to improve the professional competence and technical proficiency of all staff. ④ Implementation of measures: Dedicated supervisory bodies were established, and full-time supervisors were appointed to participate in quality management activities alongside hospital leadership. They monitored managers’ quality management performance and supervised the TQM process. Staff motivation across departments was encouraged to promote collective participation in improving medical quality. Departmental issues were identified promptly, and targeted education and training were reinforced to improve professional standards and reduce the occurrence of adverse events. The hospital also optimized the patient complaint handling process to ensure that patient feedback was promptly communicated to relevant departments for timely resolution. A reward and punishment mechanism was established: staff with consistent excellence and high efficiency received performance bonuses (10–15%) and priority consideration for excellence awards; individuals who identified significant safety hazards or proposed effective improvement suggestions were granted special bonuses ranging from 1,000 to 3,000 yuan. For minor first-time violations, 3–5% of performance pay was deducted with mandatory corrective training; major negligence resulted in a 20–50% deduction, and in severe cases, suspension of professional practice qualifications. Through this incentive and disciplinary system, staff motivation was strengthened, fostering improvements in both medical quality and service standards.

### Statistical analysis

All data analyses were performed using SPSS 29.0 statistical software. Numeration data were presented as n (%) and compared using the chi-square (χ) test. Measurement data were presented as mean ± standard deviation and analyzed using the paired-sample *t*-tests. A *P* < 0.05 indicates a statistically significant difference.

## Results

### Work management efficiency of medical staff

After implementation, the scores for hospital culture development, reward and punishment mechanisms, communication and coordination mechanisms, environmental hygiene management mechanisms, and adverse event reporting mechanisms among medical staff were higher than those before implementation (89.90 ± 1.92 vs. 95.82 ± 1.16, 89.70 ± 2.18 vs. 96.20 ± 1.21, 89.26 ± 1.32 vs. 96.32 ± 0.55, 88.82 ± 1.59 vs. 96.20 ± 1.82, 88.24 ± 2.50 vs. 96.80 ± 1.09, *t* = 18.850, 17.380, 33.500, 19.930, 18.790, *P* < 0.001). These results suggest that TQM intervention was associated with the optimization of multiple dimensions of medical staff management work efficiency (Table 1).

**TABLE 1:** Comparison of management efficiency scores of medical staff before and after implementation ($\bar{x}$± s, score).

Group	Hospital culture development	Reward and punishment mechanisms	Communication and coordination mechanisms	Environmental hygiene management mechanisms	Adverse event reporting mechanisms
Before implementation (*n* = 50)	89.90 ± 1.92	89.70 ± 2.18	89.26 ± 1.32	88.82 ± 1.59	88.24 ± 2.50
After implementation (*n* = 50)	95.82 ± 1.16	96.20 ± 1.21	96.32 ± 0.55	96.20 ± 1.82	96.80 ± 1.09
*T*	18.850	17.380	33.500	19.930	18.790
*P*	< 0.001	<0.001	<0.001	<0.001	<0.001

### Medical service quality

After implementation, the scores for clinical nursing and medical capabilities, interpersonal relationships, ethics and law, professional development, and critical thinking among medical staff were higher in contrast with those before implementation (14.12 ± 0.69 vs. 17.06 ± 1.22, 13.78 ± 0.58 vs. 17.62 ± 0.92, 14.44 ± 0.88 vs. 17.82 ± 1.22, 11.94 ± 0.42 vs. 17.78 ± 0.86, 11.32 ± 0.84 vs. 17.40 ± 0.67, t = 14.210, 25.690, 18.060, 41.410, 42.700, *P* < 0.001). These findings indicate that TQM intervention contributed to significant improvements in various dimensions of medical service quality (Table 2).

**TABLE 2:** Comparison of medical service quality scores of medical staff before and after implementation ($\bar{x}$± s, score).

Group	Clinical nursing and medical capabilities	Interpersonal relationships	Ethics and law	Professional development	Critical thinking
Before implementation (*n* = 50)	14.12 ± 0.69	13.78 ± 0.58	14.44 ± 0.88	11.94 ± 0.42	11.32 ± 0.84
After implementation (*n* = 50)	17.06 ± 1.22	17.62 ± 0.92	17.82 ± 1.22	17.78 ± 0.86	17.40 ± 0.67
*T*	14.210	25.690	18.060	41.410	42.700
*P*	< 0.001	<0.001	<0.001	<0.001	<0.001

### Satisfaction

The overall satisfaction rate of medical staff before implementation was 66% (33/50), while after implementation, it increased to 98% (49/50). The overall satisfaction of medical staff after implementation was higher in comparison to that before implementation (*P* < 0.001), suggesting a strong association between TQM intervention and improved staff satisfaction (Table 3).

**TABLE 3:** Comparison of satisfaction among medical staff before and after implementation [n (%)].

Group	Very satisfied	Satisfied	Dissatisfied	Overall satisfaction
Before implementation (*n* = 50)	14 (28.00)	19 (38.00)	17 (34.00)	33 (66.00)
After implementation (*n* = 50)	29 (58.00)	20 (40.00)	1 (2.00)	49 (98.00)
χ				17.344
*P*				<0.001

### Safety quality

Before implementation, the incidence rates of adverse events, unplanned returns to the operating room, infection events, medical complaints, and the disinfection qualification rate of medical devices were 20, 10, 10, 20, and 80%, respectively. After implementation, the corresponding rates were 2, 0, 0, 2, and 100%, respectively. After implementation, the incidence rates of adverse events, unplanned returns to the operating room, infection events, and medical complaints were lower in contrast with those before implementation (χ = 8.274, 5.263, 5.263, 8.274, *P* = 0.004, 0.022, 0.022, 0.004). Additionally, the disinfection qualification rate of medical devices was higher relative to that before implementation (χ = 11.111, *P* = 0.001). These results indicate that TQM intervention effectively improved safety quality (Table 4).

**TABLE 4:** Comparison of safety quality before and after implementation [n (%)].

Group	Incidence rate of adverse events	Unplanned returns to the operating room rate	Infection incidence rate	Medical complaint rate	Disinfection qualification rate of medical devices
Before implementation (*n* = 50)	10 (20.00)	5 (10.00)	5 (10.00)	10 (20.00)	40 (80.00)
After implementation (*n* = 50)	1 (2.00)	0 (0.00)	0 (0.00)	1 (2.00)	50 (100.00)
χ	8.274	5.263	5.263	8.274	11.111
*P*	0.004	0.022	0.022	0.004	0.001

### Medical quality

After implementation, the scores for medical safety management, medical training management, and medical hygiene supervision management were higher relative to those before implementation (13.72 ± 0.78 vs. 17.32 ± 0.65, 4.32 ± 0.55 vs. 17.64 ± 0.92, 13.96 ± 0.57 vs. 17.58 ± 1.01, *t* = 23.400, 25.700, 23.900, *P* < 0.05). These findings suggest that TQM intervention was associated with substantial optimization of overall medical quality (Table 5).

**TABLE 5:** Comparison of medical quality scores before and after implementation ($\bar{x}$ ± s, score).

Group	Medical safety management	Medical training management	Medical hygiene supervision management
Before implementation (*n* = 50)	13.72 ± 0.78	14.32 ± 0.55	13.96 ± 0.57
After implementation (*n* = 50)	17.32 ± 0.65	17.64 ± 0.92	17.58 ± 1.01
*T*	23.400	25.700	23.990
*P*	<0.001	<0.001	<0.001

## Discussion

The quality management system occupies a pivotal role in hospitals (9). To safeguard public health, ensure the safety of both patients and medical staff, enhance the quality of medical services, and establish an efficient healthcare system, quality management measures are of vital importance for maintaining a high standard of living (2). This study thoroughly evaluated the feasibility of TQM in the field of medical quality management and explored its actual impact on work efficiency.

The results of this study indicate that TQM improves efficiency through two major pathways: optimization of management processes and enhancement of staff competence. First, at the management efficiency level, improvements in communication and coordination mechanisms and adverse event reporting mechanisms (e.g., an increase in the latter’s score from 88.24 to 96.80) were correlated with a reduction in the incidence of adverse events from 20 to 2%. This suggests that TQM may facilitate a shift from post-incident response to proactive prevention by establishing an open, non-punitive safety culture that encourages staff to voluntarily report potential risks, thereby enhancing the early warning capacity and responsiveness of the management system. Second, in terms of personnel development, the simultaneous optimization of professional development (increasing from 11.94 ± 0.42 to 17.78 ± 0.86) and reward and punishment mechanisms (increasing from 89.70 ± 2.18 to 96.20 ± 1.21) effectively stimulated healthcare workers’ intrinsic motivation. This promoted a comprehensive improvement in professional competencies, such as clinical care capabilities and critical thinking, while overall staff satisfaction increased markedly from 66 to 98%. These associations indicate that TQM may play an essential role in enhancing human resource efficiency. Ultimately, these multidimensional improvements converge to drive organizational excellence. The standardization and optimization of systemic processes—such as medical safety management and training management—were also correlated with improved quantitative indicators, including a decline in infection events from 10 to 0% and an increase in the medical device disinfection pass rate from 80 to 100%. Collectively, these findings provide empirical evidence supporting the potential value of TQM in improving medical quality and safety.

The results of this study are consistent with existing research on TQM while offering more specific empirical support. First, our findings align with those of Mohammed et al. in the construction industry, who reported that TQM significantly enhances organizational effectiveness through systematic process management (10). The present study further demonstrates that this mechanism is equally applicable within the complex healthcare environment, as reflected by substantial improvements in standardized scores for medical safety management and training management processes. Data also show that TQM exerts a direct and significant impact on scientific research, community service, and the quality of graduates in higher education institutions (11). Furthermore, the application of TQM in independent nursing practice has been shown to enhance organizational performance and function as a strategic framework for evaluating and implementing sustainable improvements (12).

In the healthcare domain, previous reports have indicated that implementing a zero-tolerance policy for winged metal needles—based on TQM principles—led to significant quality improvements, including a reduction in both the use of such needles and the occurrence of needlestick injuries (8). The improvement in adverse event reporting mechanisms and the corresponding decline in adverse event incidence observed in this study further validate the effectiveness of TQM in establishing a strong safety culture and promoting a shift from reactive management to proactive prevention. Additionally, Majdi et al. confirmed that TQM interventions can drive continuous quality improvement and help achieve cost-effective, high-quality care (2). Similar patterns were identified in this study, with enhancements observed in healthcare workers’ professional competencies and multiple medical quality indicators, providing further evidence of the practical value of TQM in clinical settings. Rasha et al. emphasized that the “hard” elements of TQM (e.g., standardized processes for medical safety and training management) form the structural foundation for quality assurance, whereas the “soft” elements (e.g., incentive mechanisms and professional development) effectively activate staff initiative—together constituting essential pillars for improving internal quality performance (13). The “dual pathways” of “management process optimization” and “staff competence enhancement” revealed in this study similarly offer an integrative perspective for understanding the comprehensive mechanism of TQM.

This study has several limitations. First, the single-group pre-post test design with a small sample size (*n* = 50) drawn from a single institution, despite yielding statistically significant results, limits the generalizability of findings to other medical institutions and makes it difficult to entirely exclude potential confounding influences such as historical events or maturation effects. Second, although quantitative scales were utilized, certain data (e.g., satisfaction ratings) may be affected by social desirability bias. Third, the relatively short observation period restricts the ability to evaluate the long-term sustainability of TQM intervention effects, and its enduring benefits require further validation. In light of these limitations, future research should employ more rigorous randomized controlled trial designs, expand the sample size and institution types, and establish more reliable causal relationships to verify the universality of the TQM model. Moreover, long-term longitudinal tracking should be conducted to assess the sustained influence of TQM on employee behavior and overall hospital performance.

In summary, TQM is feasible and effective in medical quality management. It exerts a positive influence on improving healthcare workers’ efficiency, enhancing medical service quality, boosting staff satisfaction, and elevating both safety and overall medical quality. Based on these findings, hospital administrators should focus TQM implementation on key areas such as optimizing communication processes, fostering a non-punitive adverse event reporting culture, and strengthening employee professional development—serving as practical levers for driving efficiency and quality improvement. Although the initial investment in training and system development may be substantial, the demonstrated reductions in adverse events and unplanned returns to the operating room highlight TQM’s high cost-effectiveness. Furthermore, it is recommended that health policymakers consider incorporating core TQM principles—such as safety culture development and process management indicators—into hospital evaluation systems, thereby guiding medical institutions to transition from scale-oriented growth to quality-driven development.

## Data Availability

The original contributions presented in this study are included in the article/supplementary material, further inquiries can be directed to the corresponding author.
